# Development of a Microvessel Density Gene Signature and Its Application in Precision Medicine

**DOI:** 10.1158/2767-9764.CRC-24-0403

**Published:** 2025-03-05

**Authors:** Megumi Kuronishi, Yoichi Ozawa, Takayuki Kimura, Shuyu Dan Li, Yu Kato

**Affiliations:** 1 Tsukuba Research Laboratories, Eisai Co., Ltd., Tsukuba, Japan.; 2 Eisai Inc., Nutley, New Jersey.

## Abstract

Combination therapy with antiangiogenic drugs and immune checkpoint inhibitors has shown enhanced clinical activity and has been approved for the treatment of multiple tumor types. Despite extensive research, predictive biomarkers for combination therapy remain poorly understood. Microvessel density (MVD), a surrogate marker for aberrant angiogenesis measured by IHC, has been associated with response to monotherapy with antiangiogenic inhibitors. However, obtaining tumor tissue with a sufficient mass for IHC analysis is not always practical, and IHC-based MVD measurements are unavailable in large public datasets. In this study, we developed an MVD gene score based on RNA sequencing data that reflects MVD by using RNA sequencing and MVD measured by IHC in 12 mouse syngeneic tumor models. We explored the relationship between the MVD gene score and a gene signature, predicting the response to anti–PD-1 therapy in mouse and human tumor datasets. The MVD gene score correlated with the antitumor activity of lenvatinib, a multiple tyrosine kinase inhibitor mainly targeting VEGFRs and FGFRs, in mouse tumor models, and MVD measured by IHC in commercially available human formalin-fixed, paraffin-embedded tumor samples. Tumor types in The Cancer Genome Atlas were classified into four subgroups based on the MVD gene score and T cell–inflamed gene expression profile, which were correlated with clinical indications for treatment. In conclusion, the newly developed MVD gene score enables the estimation of MVD in large public datasets in which IHC data are unavailable and has potential clinical utility together with the T cell–inflamed gene expression profile to characterize tumors of patients for precision medicine.

**Significance:**

A novel gene signature for MVD was developed. This MVD gene score enables the estimation of MVD, reflecting the sensitivity to antiangiogenic inhibitors, in transcriptomic datasets. We demonstrated the utility of the MVD gene score together with a T cell–inflamed gene signature for potential future use as a clinical biomarker.

## Introduction

The tumor microenvironment (TME) is composed of cancer cells and other surrounding nonmalignant cells, including tumor endothelial cells (EC), immune cells, cancer-associated fibroblasts, and the extracellular matrix, which collectively play critical roles in the pathogenesis of cancer (1). In addition to cancer cell–intrinsic factors, many TME-related factors, such as tumor vasculature and antitumor immunity, are associated with sensitivity and resistance to cancer therapies (1).

Aberrant angiogenesis is a hallmark of cancer in which the VEGF signaling pathway plays an essential role (2). VEGF signaling also contributes to an immunosuppressive TME, making antiangiogenic inhibitors successful combination partners with immune checkpoint inhibitors (ICI) in some indications, including renal cell, hepatocellular, and endometrial carcinomas (3–6). Lenvatinib (LEN) is a multitargeted receptor tyrosine kinase inhibitor mainly targeting VEGFRs 1 to 3 and FGFRs 1 to 4 (7–9). Preclinical studies have shown that LEN exerts antitumor effects by inhibiting aberrant tumor angiogenesis and immunomodulatory activity (8–11). Combination treatment with anti–PD-1 shows enhanced antitumor efficacy, and LEN in combination with pembrolizumab has been approved for the treatment of advanced renal cell (4) and endometrial carcinomas (6).

Despite extensive research, predictive biomarkers for ICIs plus angiogenic inhibitors remain incompletely elucidated. Microvessel density (MVD), a surrogate marker of aberrant angiogenesis measured by IHC, has been associated with the response to antiangiogenic inhibitors, including LEN (9, 12–14). Additionally, T cell–inflamed, immune “hot” tumors have been associated with greater response to ICIs, and gene signatures surrogating preexisting adaptive intratumor immunity such as T cell–inflamed gene expression profile (Tcell_inf_GEP) have been developed to predict the response to anti–PD-1 therapies including pembrolizumab (15–17).

Based on previous findings, we hypothesized that MVD and Tcell_inf_GEP are also associated with the response to combination treatment. However, testing this hypothesis has been difficult because of lack of broad availability of IHC-based MVD data. Obtaining tumor tissue with a sufficient mass for IHC analysis is not always practical, and gene signatures are complementarily used as surrogate markers of pathway activity and certain phenotypes. In addition, large public cancer datasets such as The Cancer Genome Atlas (TCGA; ref. 18) allow us to explore the relationship between gene signatures across various tumor types. Previously developed angiogenesis- and EC-related gene signatures contain genes that were highly expressed in ECs compared with other cell types (19, 20), downregulated by anti-VEGF therapies (21, 22), and co-expressed with well-known EC marker genes or angiogenesis-related genes (16, 23–25). However, gene signatures developed to reflect MVD from bulk RNA sequencing (RNA-seq) of tumor samples have been unavailable so far.

In this study, we evaluated the correlation between MVD measured by IHC [MVD(IHC)] and the antitumor activity of LEN in 12 mouse syngeneic tumor models. Next, we developed an MVD gene score based on single-cell RNA-seq (scRNA-seq) data and paired bulk RNA-seq and MVD(IHC) from mouse tumor models. We validated the correlation between MVD gene score and MVD(IHC) in bulk RNA-seq and MVD(IHC) data from human tumor samples, enabling the estimation of MVD(IHC) in external datasets in which IHC data are unavailable. We then assessed the baseline characteristics of 12 mouse syngeneic models in terms of MVD(IHC) and Tcell_inf_GEP, potential predictive biomarkers of antiangiogenic inhibitors and ICIs, and grouped them into subgroups defined by the two TME-related biomarkers. We also explored the relationship between these gene signatures in a large human tumor dataset (TCGA). We further conducted *in vivo* drug efficacy and RNA-seq experiments in 12 mouse syngeneic tumor models to assess the relationship between tumor subgroups and the efficacy of LEN, anti–PD-1, and combination therapy.

## Materials and Methods

### Mouse tumor cell lines and animals

The sources of cancer cells and mice used in this study are summarized in Supplementary Table S1. For the RAG model, we used *in vivo* adapted RAG cells, as previously described (26).

### Human tumor tissue samples

Commercially available formalin-fixed, paraffin-embedded (FFPE) human tumor tissue samples from patients who provided written informed consent were obtained by vendors. These samples were purchased and used in this study. The experiments using human samples were conducted in accordance with the Declaration of Helsinki and Ethical Guidelines for Medical and Biological Research Involving Human Subjects in Japan and approved by the Research Ethics Committee of Eisai Co., Ltd. The tumor types and number of samples used in this study are summarized in Supplementary Table S2.

### Antitumor activity, tumor sampling, and calculation of Δ*T*/*C*

Cancer cells were subcutaneously implanted into the right flank of 7-week-old mice. When the tumors reached a size of approximately 100 mm^3^, the mice were randomized into treatment groups [no treatment (NT), LEN, anti–PD-1, and LEN plus anti–PD-1; 8 mice per group], and LEN (dissolved in 3 mmol/L HCl, 10 mg/kg) was administered daily by oral gavage. Anti–PD-1 (200 μg/head) was intraperitoneally administered twice weekly. Tumor volume (TV) was calculated using the formula TV (mm^3^) = 0.5 × length (mm) × width^2^ (mm^2^). TV was monitored up to 2,000 mm^3^, and mice were sacrificed when TV was >2,000 mm^3^ as a humane endpoint. No severe loss in body weight was observed (Supplementary Fig. S5). The day on which treatment was initiated was designated as day 1. For RNA-seq and IHC analyses, tumor sampling was conducted when the TVs reached approximately 100 mm^3^ for the treatment-naïve tumors and on day 8 for the comparison of the NT and LEN-treated groups.

The values of antitumor activity (Δ*T*/*C*) were calculated using the formula Δ*T*/Δ*C* × 100, in which Δ*T* and Δ*C* are the changes in the mean TVs for drug-treated and untreated control groups, respectively. When the TVs decreased from the initial TVs, Δ*T*/*C* values were calculated as (*T*_*t*_ − *T*_1_)/*T*_1_ × 100, in which *T*_*t*_ and *T*_1_ are the mean TVs of the drug-treated group on day *t* after the start of treatment and day 1, respectively. All animal experiments were performed in accordance with guidelines approved by the Institutional Animal Care and Use Committee of Eisai Co., Ltd.

### RNA-seq

RNA-seq of mouse and human tumor tissues was performed using the NovaSeq 6000 and NextSeq 550 platforms (Illumina). Sequencing reads were subjected to quality assessment using FastQC v0.11.5 (RRID: SCR_014583). Illumina adapter sequences and low-quality bases were trimmed using Trimmomatic software v0.36 (RRID: SCR_011848). Filtered reads were mapped to the mouse or human genome (GRCh38, mm10, and Ensembl 88) using STAR v2.5.2b (RRID: SCR_004463). Gene expression levels were estimated in transcripts per million for all samples using RSEM v1.2.31 (RRID: SCR_000262). Samples that did not pass quality control were excluded from the analysis.

### MVD(IHC) calculation

Using the same samples as those used for RNA-seq, CD31 in frozen mouse tumor tissues and CD34 in human FFPE tumor tissues were stained with anti–mouse CD31 (clone SZ31, Dianova) or anti–human CD34 (clone QBEnd/10, Leica Biosystems) antibodies, respectively. When human tumor samples were stained with the anti-CD31 antibody, both microvessels and lymphocytes were found to be significantly stained. Therefore, we used an anti–human CD34 antibody to detect microvessels in human tumor samples based on (27). The whole tumor area of the stained samples was scanned using NanoZoomer S60 (Hamamatsu Photonics), and MVD(IHC) was calculated by dividing the number of CD31- or CD34-positive blood vessels by the tumor area.

### Gene selection

Hepa 1-6 tumor samples from six different mice were collected for the scRNA-seq experiment at the timepoint when the antitumor effect of LEN is distinctly observed, specifically on day 15 of antitumor efficacy studies, which corresponds to day 25 after tumor inoculation. The mean TV and SD were 559 and 309 mm^3^, respectively. Tumor samples were pooled and dissociated with a Tumor Dissociation Kit and a gentleMACS Dissociator (Miltenyi Biotec), and scRNA-seq was conducted for the tumor cells dissociated using a Chromium Controller (10× Genomics). After filtering low-quality cells, 5,537 cells were used for the analysis. Fourteen cell clusters were classified based on their expression profiles by graph-based clustering using the Loupe Browser v.6.1.0 (10× Genomics, RRID: SCR_018555) and annotated based on the expression of known cell marker genes from previous studies (10, 28–30). Genes that were more highly expressed in the EC cluster than in the other clusters (adjusted *P* < 0.05 and log_2_ fold change >1 by the Loupe Browser’s globally distinguishing feature comparison with the Benjamini–Hochberg procedure) were selected as candidate genes for the MVD gene score. Pearson correlation coefficients between MVD(IHC) and logarithm-transformed expression values of the EC-related genes in treatment-naïve tumors of 12 mouse syngeneic tumor models were computed. The top 20 correlated genes and well-known angiogenic markers were further selected based on known associations with vasculature in the literature and their specificity of expression in EC in scRNA-seq data from the Hepa 1-6 tumor model. Using the Loupe Browser, we confirmed that the six selected genes were predominantly expressed in the EC cluster of the scRNA-seq data from the Hepa 1-6 tumor model (Supplementary Fig. S2C). The MVD gene score was calculated as the average of logarithm-transformed gene expression values.

### TCGA dataset

Gene expression data were downloaded from cBioPortal (31, 32). Cell-type deconvolution data were downloaded from Data4Cure (https://www.data4cure.com/).

### Statistical analysis

Statistical analyses were performed using Python v3.9.6 (RRID: SCR_008394) or GraphPad Prism v9.0.2 (RRID: SCR_002798). The Pearson correlation coefficient was used to compute the correlation between the MVD gene score and MVD(IHC). A Welch *t* test was used for two-group comparisons. *P* values less than 0.05 were considered significant. The Benjamini–Hochberg procedure was used for multiple testing corrections.

### Data availability

The mouse sequence data generated in this study are publicly available in the Gene Expression Omnibus (RRID: SCR_005012) under accession number GSE281579. TCGA data analyzed in this study were obtained from cBioPortal. The human sequence data generated in this study are not publicly available because of patient privacy requirements but are available upon reasonable request to the corresponding author.

## Results

### Relationship between the antitumor activity of LEN and MVD in 12 mouse syngeneic tumor models

To assess the association between the antitumor activity of LEN and MVD(IHC) in different tumor models, we first evaluated *in vivo* antitumor activity of LEN in 12 mouse syngeneic tumor models (Supplementary Table S1).

**Figure 1:**
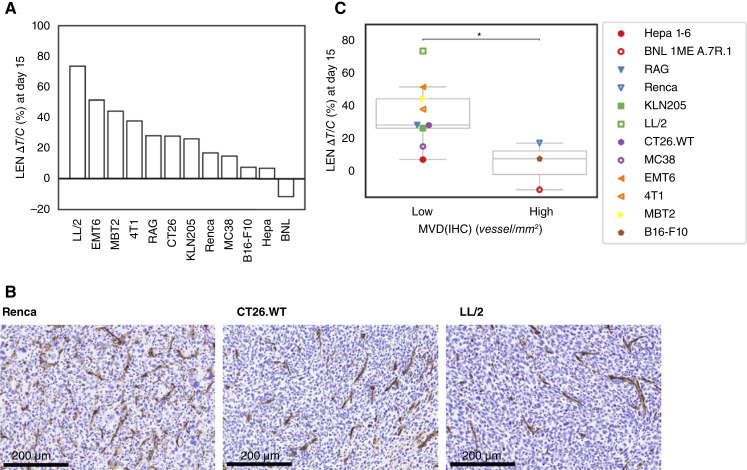
Antitumor activity of LEN in 12 mouse syngeneic tumor models and relationship with MVD(IHC). **A,** Antitumor activities of LEN are shown as Δ*T*/*C* values at day 15. Smaller Δ*T*/*C* values indicate a greater tumor growth inhibition and hence greater antitumor activities of LEN. **B,** IHC images of CD31 expression in representative models. **C,** Relationship between LEN Δ*T*/*C* at day 15 and MVD(IHC) of treatment-naïve tumor samples. Twelve models were classified as MVD(IHC)-high or MVD(IHC)-low by top quartile of mean MVD(IHC) values of each tumor model (*n* = 5). * indicates *P* < 0.05 by Welch *t*-test. Tumor models of the same tumor type were shown in the same color: red, HCC; blue, renal cell carcinoma; green, lung cancer; purple, colorectal cancer; orange, breast cancer; yellow, bladder cancer; brown: melanoma. BNL, BNL 1ME A.7R.1; CT26, CT26.WT; Hepa, Hepa 1-6.

Cancer cells were subcutaneously transplanted into mice, and treatment with LEN (10 mg/kg, once daily) was initiated when the TVs reached approximately 100 mm^3^. LEN monotherapy showed varying antitumor activities in the models, ranging from tumor reduction to a slight inhibition of tumor growth (Fig. 1A; Supplementary Fig. S1A). Next, to test the association between MVD(IHC) and sensitivity to LEN, MVD(IHC) was measured by IHC staining of blood vessels with anti-CD31 antibodies in treatment-naïve tumors (*n* = 5 for each tumor model, Supplementary Fig. S1B). Figure 1B shows representative images of CD31 staining from a different experiment. Twelve models were classified as MVD(IHC)-high or MVD(IHC)-low by top quartile of mean MVD(IHC) values of each tumor model, and LEN showed stronger antitumor activity measured as Δ*T*/*C* at day 15 in tumor models with higher MVD(IHC) values (*P* = 0.038, Welch *t* test; Fig. 1C). These results suggest that a high MVD(IHC) indicates the sensitivity of tumor models to LEN, although LEN exerts antitumor activity in various tumor models.

### Novel gene expression signature that correlates with MVD(IHC)

**Figure 2:**
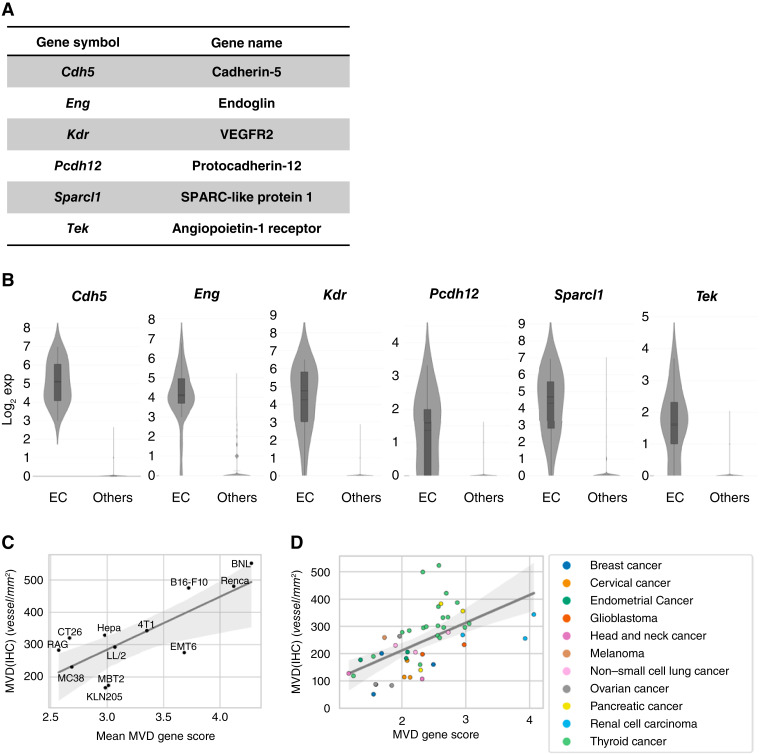
MVD gene score development in preclinical study. **A,** Six MVD-related genes. **B,** Expression levels of the six genes in the vascular endothelial cell cluster and other clusters using the scRNA-seq dataset of Hepa 1-6 tumors. **C,** Relationship between the MVD gene score and MVD(IHC) in treatment-naïve tumors of 12 mouse syngeneic tumor models. *r* = 0.77 (*P* = 0.0032) by Pearson correlation coefficient. **D,** Relationship between the MVD gene score and MVD(IHC) in human samples. *r* = 0.57 (*P* = 3.3 × 10^−5^) by Pearson correlation coefficient. MVD(IHC) and the MVD gene score computed from RNA-seq data were compared using commercially available FFPE samples of human tumors. The colors at each point represent the tumor type in the sample, as shown in the legend.

We further developed a gene signature based on RNA-seq data (MVD gene score) that correlates with MVD(IHC), enabling the estimation of MVD in external datasets in which IHC data are unavailable. To define this gene signature, we selected genes in a stepwise manner. First, we performed scRNA-seq of the Hepa 1-6 model to identify genes highly expressed in tumor-associated ECs (Supplementary Figs. S2A and S2B). LEN has been approved for the treatment of patients with hepatocellular carcinoma (33). Hepa 1-6, a mouse tumor model of hepatocellular carcinoma, is sensitive to LEN monotherapy and combination therapy with anti–PD-1 (10). Therefore, we utilized Hepa 1-6 as a tumor model for scRNA-seq analysis. Subsequently, we expanded the analysis to a panel of 12 syngeneic tumor models including Hepa 1-6. A total of 365 genes that showed higher expression in the EC cluster than in the other clusters (adjusted *P* < 0.05 and log_2_ fold change >1) were then selected as EC-related genes (Supplementary Table S3). RNA-seq experiments were performed using treatment-naïve tumors from 12 mouse syngeneic tumor models, each approximately 100 mm^3^ in size, to evaluate the baseline characteristics of the tumors in terms of MVD. The top 20 genes with the highest correlation coefficients between MVD(IHC) and expression levels were selected. Among the MVD-correlated genes with known associations with the vasculature and three well-known EC markers (*Kdr*, *Cdh5*, and *Tek*; refs. 34–37), six genes were selected based on the specificity of their expression in the EC cluster in scRNA-seq data by manual inspection of t-distributed stochastic neighbor embedding plots (Supplementary Fig. S2C). These six genes (Fig. 2A) were highly expressed in the EC cluster compared with other clusters (Fig. 2B). The MVD gene score was defined as the average of the expression values of the six genes on a logarithmic scale. MVD(IHC) and MVD gene score were significantly correlated in treatment-naïve tumors from the 12 mouse syngeneic tumor models (Pearson correlation coefficient (*r*) = 0.77; *P* = 0.0032; Fig. 2C).

To verify that the MVD gene score also correlates with MVD(IHC) in human samples, we performed RNA-seq and blood vessel staining with the human EC marker CD34 by IHC using commercially available FFPE samples of human tumors (Supplementary Table S2) and computed the MVD gene score and MVD(IHC). The MVD gene score and MVD(IHC) showed a significant correlation in human tumor samples (Fig. 2D; *r* = 0.57; *P* = 3.3 × 10^−5^), suggesting that the MVD gene score originally developed using the mouse tumor dataset also reflects MVD(IHC) in human samples. Furthermore, to examine the difference between MVD gene score and other angiogenesis-related gene signatures used in clinical biomarker analyses (16, 22, 25), we compared the correlation coefficient of each gene signature and MVD(IHC) in commercially available human FFPE tumor samples (Supplementary Fig. S3A) and the EC score estimated by cell-type deconvolution analysis using TCGA dataset (Supplementary Fig. S3B). The MVD gene score showed a stronger correlation with MVD(IHC) and the EC score than other angiogenesis-related gene signatures, indicating that the MVD gene score reflects microvessels rather than angiogenic activity itself.

**Figure 3:**
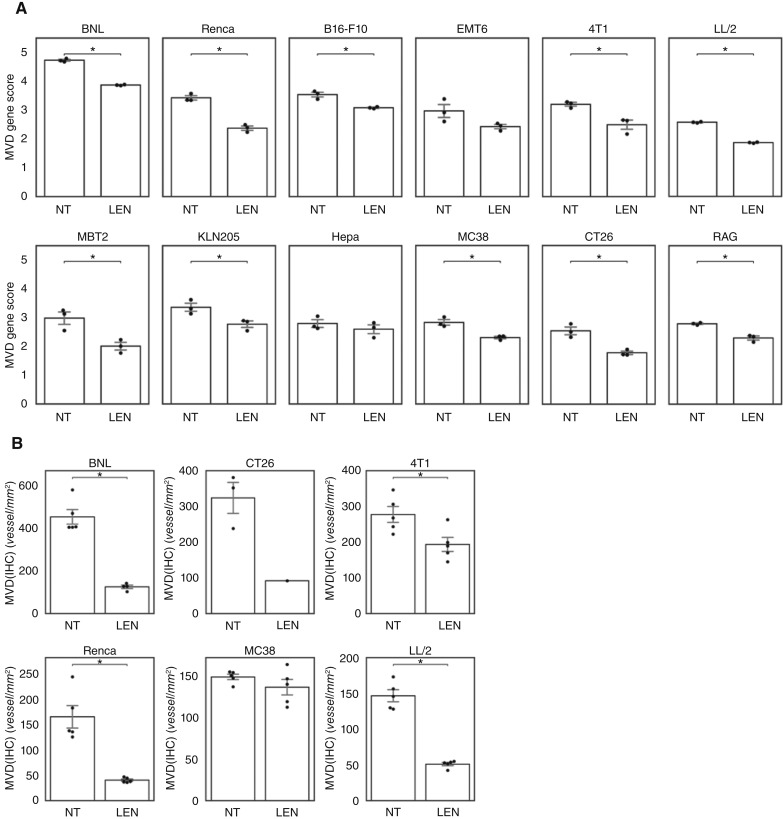
Changes in MVD gene score, TV, and MVD(IHC) of 12 mouse syngeneic tumor models with or without LEN treatment. RNA-seq analysis and MVD staining of CD31 by IHC were performed, and TV was measured for the tumors collected after the administration of LEN for 1 week or without treatment (NT). **A,** MVD gene score of the 12 models with or without LEN treatment on day 8. Data are shown as mean ± SEM. * indicates adjusted *P* < 0.05 by the Welch *t* test with the Benjamini–Hochberg procedure. **B,** MVD(IHC) of representative tumor models treated with LEN for 1 week. Data are shown as mean ± SEM. * indicates adjusted *P* < 0.05 by the Welch *t* test with the Benjamini–Hochberg procedure except for CT26. BNL, BNL 1ME A.7R.1; CT26, CT26.WT; Hepa, Hepa 1-6.

LEN reduces MVD(IHC) in tumors because of its antiangiogenic activity. Next, we conducted RNA-seq experiments on tumors from mice treated with LEN for 1 week to examine whether the MVD gene score could reflect the decrease in blood vessels caused by LEN. When compared with the NT group, the MVD gene score decreased in 10 tumor models in the LEN-treated group (Fig. 3A, adjusted *P* < 0.05), suggesting that the MVD gene score reflects the decrease in blood vessels induced by the antiangiogenic effect of LEN treatment. The MVD(IHC) of the representative models also decreased in the LEN-treated groups (Fig. 3B). We also confirmed that the MVD gene score was associated with the antitumor efficacy of LEN. Based on the top quartile of the mean MVD gene score of the 12 tumor models, the MVD gene score–high and –low groups were the same as the MVD(IHC)-high and -low groups. The Δ*T*/*C* of LEN at day 15 was correlated with the MVD gene score calculated from RNA-seq data of treatment-naïve tumors from 12 mouse syngeneic tumor models (high vs. low groups, *P* = 0.038; Fig. 1C).

These results suggest that the MVD gene score correlates with MVD(IHC) and the antitumor effects of LEN. Furthermore, the MVD gene score may reflect a decrease in blood vessels induced by the antiangiogenic effects of LEN.

### Relationship between MVD and Tcell_inf_GEP signatures in mouse and human tumor datasets

Many studies have shown that antiangiogenic inhibitors in combination with ICIs exhibit enhanced antitumor activity (3–6). Tcell_inf_GEP is another gene signature associated with sensitivity to anti–PD-1 treatment (15). To explore the baseline characteristics of the 12 mouse syngeneic tumor models consisting of multiple tumor types, we compared the known biomarkers of each monotherapy, MVD(IHC) and Tcell_inf_GEP signatures, in treatment-naïve tumors. We then extended the comparison with a human tumor dataset using the MVD gene score.

**Figure 4:**
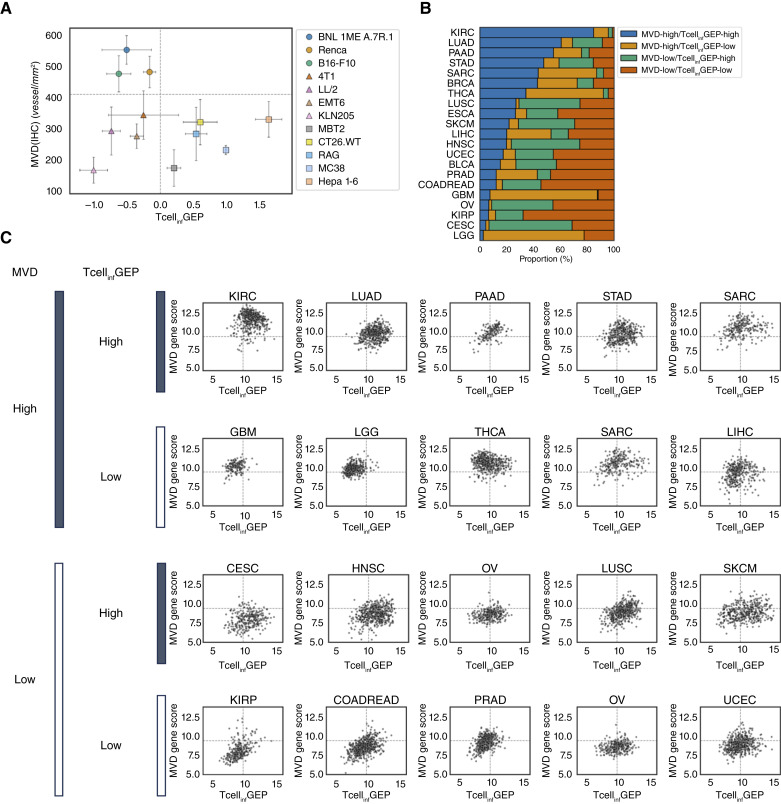
Relationship between MVD gene score and Tcell_inf_GEP in mouse syngeneic tumor models and human pan-cancer dataset. **A,** Relationship between MVD(IHC) and Tcell_inf_GEP of baseline tumors in 12 mouse syngeneic models. Data are shown as mean ± SD [*n* = 5 for MVD(IHC); *n* = 3 except for MC38 (*n* = 2) for Tcell_inf_GEP]. **B,** Proportion of samples in each subgroup defined by median values of the MVD gene score and Tcell_inf_GEP in all samples. **C,** MVD gene score and Tcell_inf_GEP within each major solid tumor type computed using gene expression data from TCGA pan-cancer dataset. Dotted lines represent the median value of each gene signature in all samples. BLCA, bladder urothelial carcinoma; BRCA, breast invasive carcinoma; CESC, cervical squamous cell carcinoma and endocervical adenocarcinoma; COADREAD, colon adenocarcinoma and rectal adenocarcinoma; ESCA, esophageal carcinoma; GBM, glioblastoma multiforme; HNSC, head and neck squamous cell carcinoma; KICH, kidney chromophobe; KIRP, kidney renal papillary cell carcinoma; LGG, brain lower-grade glioma; LUAD, lung adenocarcinoma; LUSC, lung squamous cell carcinoma; OV, ovarian serous cystadenocarcinoma; PAAD, pancreatic adenocarcinoma; PRAD, prostate adenocarcinoma; SARC, sarcoma; SKCM, skin cutaneous melanoma; STAD, stomach adenocarcinoma; THCA, thyroid carcinoma; UCEC, uterine corpus endometrial carcinoma.

Based on the relative activation or suppression of MVD and Tcell_inf_GEP in the models, the mouse tumor models were categorized into three subgroups: MVD-high/Tcell_inf_GEP-low (BNL 1ME A.7R.1, Renca, and B16-F10), MVD-low/Tcell_inf_GEP-high (Hepa 1-6, MC38, CT26.WT, RAG, and MBT2), and MVD-low/Tcell_inf_GEP-low (KLN205, LL/2, EMT6, and 4T1; Fig. 4A). MVD and Tcell_inf_GEP of individual tumors are shown in Supplementary Figs. S1B and S4A. There were no MVD-high/Tcell_inf_GEP-high mouse tumor models among the 12 models. Similarly, the relationship between the MVD gene score and Tcell_inf_GEP in human tumor samples was investigated using gene expression data from TCGA pan-cancer cohort. The distribution of the MVD gene score and Tcell_inf_GEP varied among tumor types (Supplementary Fig. S4B and S4C). Samples of each tumor type were divided into subgroups based on the median values of the MVD gene score and Tcell_inf_GEP in the entire TCGA pan-cancer cohort, and the proportion of samples classified in each subgroup is shown in Fig. 4B. Unlike the mouse tumor models, several tumor types showed a higher proportion of MVD-high/Tcell_inf_GEP-high samples, and kidney renal clear-cell carcinoma (KIRC) had the highest proportion. The five major cancer types with the highest percentages in each subgroup are shown in Fig. 4C. There were two tumor types in which most samples were categorized into one subgroup (KIRC and glioblastoma multiforme) and tumor types with broader ranges of MVD gene score and Tcell_inf_GEP, such as skin cutaneous melanoma and liver hepatocellular carcinoma (LIHC). These results suggested that tumor types showed distinct relationships between the MVD gene score and Tcell_inf_GEP and demonstrated the usefulness of the MVD gene score, which enabled such characterization in a large public dataset of human tumor samples.

### Antitumor activity of LEN in combination with anti–PD-1 in 12 mouse syngeneic tumor models

**Figure 5:**
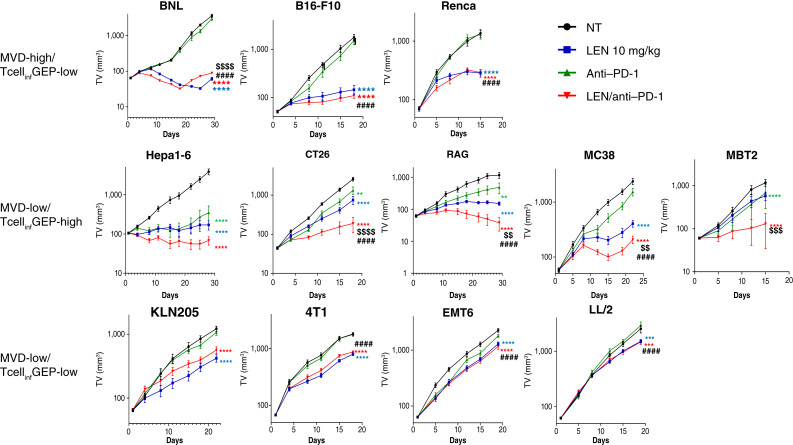
Antitumor activity of LEN plus anti–PD-1 antibody in 12 mouse syngeneic tumor models. TVs of mice with NT, LEN 10 mg/kg, anti–PD-1 antibody at 200 μg/head, and their combination groups. Data are shown as mean ± SEM. **, *P* < 0.01; ***, *P* < 0.001; ****, *P* < 0.0001 vs. NT by repeated measured Dunnett multiple comparisons using logarithm-transformed values. Asterisks are colored according to treatment groups as indicated in the legend. $$, *P* < 0.01; $$$, *P* < 0.001; $$$$, *P* < 0.0001 vs. LEN alone: ####, *P* < 0.0001 vs. anti–PD-1 antibody alone by repeated measured Dunnett multiple comparisons using logarithm-transformed values. BNL, BNL 1ME A.7R.1; CT26, CT26.WT.

MVD(IHC) and Tcell_inf_GEP measured in tumors have been associated with treatment response to LEN and anti–PD-1, respectively (9, 15). To characterize the relationship between tumor subgroups based on TME-related biomarkers and different sensitivities to treatments across tumor types, we investigated the *in vivo* antitumor activity of LEN (10 mg/kg, once daily), anti–PD-1 (200 μg/head, twice weekly), and their combination in 12 mouse syngeneic tumor models. Combination treatment with LEN and anti–PD-1 inhibited tumor growth compared with the untreated control in all 12 models, without any significant changes in body weight (Fig. 5; Supplementary Fig. S5). Enhanced antitumor activity by the combination treatment, measured as statistically significant suppression of TV compared with monotherapy, was observed in MC38, CT26.WT, and RAG, all of which were MVD-low/Tcell_inf_GEP-high models (*P* < 0.05).

Compared with the other subtypes, the MVD-high/Tcell_inf_GEP-low models were more sensitive to LEN monotherapy. MVD-low/Tcell_inf_GEP-high models were more sensitive to anti–PD-1 monotherapy, and enhanced antitumor effects were observed with combination treatment. The MVD-low/Tcell_inf_GEP-low models were relatively resistant to monotherapies, and no or little enhancement of the antitumor effects by combination treatment was observed. These results suggest that the baseline characteristics of MVD and Tcell_inf_GEP in tumors were indicative of the response to LEN, anti–PD-1, and their combination treatment.

## Discussion

We previously reported that xenograft tumor models sensitive to LEN showed higher MVD(IHC) than relatively resistant models (9). In the present study, we developed an MVD gene score based on the expression levels of six MVD-related genes. The MVD gene score computed from RNA-seq data of treatment-naïve tumors of 12 mouse syngeneic models correlated with the MVD(IHC) measured in matched tumor samples (Fig. 2C) and the antitumor activity of LEN (Fig. 1C). The MVD gene score correlated with MVD(IHC) in human tumor samples (Fig. 2D), indicating that the MVD gene score originally developed using a mouse tumor dataset also reflects MVD(IHC) in human datasets.

One drawback of using IHC-based measurements such as MVD(IHC) is analytic variability (38, 39). Our MVD gene score uses the gene expression values of multiple genes from RNA-seq experiments, making it more robust against such variabilities. Another merit of the MVD gene score is that it was obtained using scRNA-seq data from the tumor tissue. We selected genes that were highly expressed in tumor ECs compared with other cell types in tumor samples. This reduced the possibility of including genes that are expressed not only in ECs but also in other cancer or stromal cells in the TME. We further tested the correlation between the expression levels of tumor EC–related genes from bulk RNA-seq and MVD(IHC) in 12 mouse syngeneic tumor models. This approach allowed us to select a unique gene set that was specific to tumor ECs and indeed correlated with MVD(IHC). In addition, using gene signatures, we can assess the MVD in each sample of a large tumor dataset for which gene expression data are available.

We compared the MVD gene score with other angiogenic gene signatures used in clinical biomarker analyses. The IMmotion150 angiogenic signature was derived from genes that had expression levels changed in response to a VEGF blockade in preclinical tumor models and biopsies from patients treated with anti-VEGF mAbs (22). The JAVELIN Renal 101 angiogenic signature was developed from one of the co-expression network modules enriched with angiogenesis-related genes and was associated with response to sunitinib (25). Merck’s angiogenic signature was selected as genes co-expressed with known angiogenic genes, *KDR*, *TIE1*, *TEK*, and *CD34*, in public pan-cancer genomic datasets (16). Our MVD gene score focuses on the EC component of angiogenesis and its correlation with MVD, as measured by IHC. The MVD gene score is more appropriate to specifically reflect MVD instead of angiogenesis in general. The difference between the MVD signature developed in this study and previously developed angiogenic gene signatures was evident that the MVD signature had a stronger correlation with MVD(IHC) in human FFPE tumor tissue samples (Supplementary Fig. S3A) and the estimated ECs in TCGA tumor samples (Supplementary Fig. S3B).

Using the MVD gene score, we grouped human tumor samples from TCGA and 12 mouse syngeneic tumor models into subtypes based on MVD and Tcell_inf_GEP signatures. Based on the results of antitumor activities of LEN and anti–PD-1 in the mouse models, each subgroup showed different sensitivities to treatments. MVD-high/Tcell_inf_GEP-low models were sensitive to LEN, MVD-low/Tcell_inf_GEP-high models were sensitive to anti–PD-1 and combination treatment, and MVD-low/Tcell_inf_GEP-low models were relatively resistant to all treatments including combination treatment. This was consistent with the previous study that reported an association between murine Tcell_inf_GEP and sensitivity to monotreatment of anti–PD-1 and combination treatment with LEN (17). In TCGA dataset, MVD-high/Tcell_inf_GEP-low tumor types were enriched for tumor types in which angiogenic inhibitors as monotherapy can be used as standard systemic therapies (i.e., glioblastoma multiforme, thyroid carcinoma, sarcoma, and LIHC; refs. 33, 40–43), supporting the hypothesis that tumors with higher MVD are more susceptible to antiangiogenic therapies, and angiogenic inhibitors as monotherapy are effective. MVD-high/Tcell_inf_GEP-high tumor types were enriched for those in which combination therapies, including ICI plus antiangiogenic therapies, can be used as standard treatments (i.e., KIRC and lung adenocarcinoma; refs. 4, 44–48). The same is true for MVD-low/Tcell_inf_GEP-high tumor types (i.e., head and neck squamous cell carcinoma, lung squamous cell carcinoma, and skin cutaneous melanoma; refs. 45–47, 49–53). This is consistent with previous findings that preexisting antitumor immunity predicts the response to ICIs (54); however, by adding the MVD gene score as another axis, the different subgroups between these tumor types were highlighted.

Combination therapy with ICIs and angiogenic inhibitors is used in the first-line setting for KIRC (4, 55, 56) and LIHC (5). In our analysis of TCGA dataset, the percentage of samples with MVD-high/Tcell_inf_GEP-high was the highest in KIRC. In the LIHC group, the percentage of samples with MVD-high/Tcell_inf_GEP-low was the highest. Samples in the LIHC group were not exclusively in one subgroup and were more scattered. Although we could not directly test the association between MVD or Tcell_inf_GEP scores and clinical outcomes in LIHC treated with a combination of ICI and antiangiogenic drugs, genomic analysis in the IMbrave150 registration trial of atezolizumab and bevacizumab has delineated preexisting tumor immunity and high expression of VEGF receptors was associated with improved clinical outcomes (57), suggesting that tumors with MVD-high/Tcell_inf_GEP-high are likely to be more sensitive to combination treatment. Additionally, in a recent clinical biomarker study of LEN and pembrolizumab combination in endometrial cancer, patients with MVD-high/Tcell_inf_GEP-high scores demonstrated a higher objective response rate than those with tumors in other subgroups (58). Furthermore, the enhanced antitumor activity of LEN and anti–PD-1 in mouse syngeneic tumor models in the MVD-low/Tcell_inf_GEP-high group supports the possibility that combination therapy may be effective in this subgroup.

One major difference between the mouse models and TCGA dataset was the absence of the MVD-high/Tcell_inf_GEP-high subgroup in the mouse tumor models. This may be due to the limited number of mouse tumor models used in this study compared with the heterogeneity of human tumors. This problem could be solved by increasing the variety of mouse models used in these studies. Given that not all cancer cell lines can grow *in vivo*, it is also possible that mouse cancer cells that are highly dependent on tumor angiogenesis and possess immunogenic properties are more likely to be eliminated through *in vitro* culture and by the immune systems of host mice and are thus unsuitable for *in vivo* tumor models. Despite this disagreement, MVD and Tcell_inf_GEP are useful for delineating the distinct characteristics of each tumor model and tumor type and provide insights for further investigation. For example, an individual tumor of a specific tumor type with high MVD/Tcell_inf_GEP could be sensitive to combination therapy with ICI and angiogenic inhibitors, even though the tumor type itself was not associated with the MVD-high/Tcell_inf_GEP-high subgroup. It is also possible that the factors associated with resistance to ICI and angiogenic inhibitors will be revealed through further characterization of the MVD-low/Tcell_inf_GEP-low tumor subtype. Our analyses demonstrated that the gene signatures enabled us to test our hypotheses about clinical indications using large public datasets.

We have previously reported that LEN exerts antitumor activity via antiangiogenic and immunomodulatory effects (10, 11). Although the MVD gene score reflects the primary targets of LEN, the abundance of tumor microvessels, other mechanisms may also contribute to LEN sensitivity. For instance, in our analysis of the MVD gene score in the Hepa 1-6 tumors, the difference between the NT and LEN-treated groups was found to be not statistically significant, despite a strong response to LEN based on tumor size in the Hepa 1-6 model (Fig. 3A). In our previous study using the Hepa 1-6 model, LEN demonstrated antitumor activity in both immunodeficient and immunocompetent mice, with a greater activity observed in the latter (10), indicating that not only antiangiogenic activity but also immunomodulatory effects play an important role in the antitumor activity of LEN in the Hepa 1-6 model.

Recently, Uhlik and colleagues (59) reported a similar framework for classifying tumor transcriptomic profiles into four TME subtypes in terms of angiogenesis and immune activities and showed enriched objective response rates to either ICI-containing or angiogenic inhibitor–containing treatment for biomarker-positive populations in gastric cancer, ovarian cancer, and melanoma cohorts. These findings support our hypothesis that tumor angiogenesis and antitumor immune activity in pretreatment settings can predict treatment sensitivity.

This study had several limitations. One was that the clinical outcomes of LEN and ICI were unavailable for TCGA dataset. Further studies using a matched dataset of gene expression and clinical outcome information are important to validate the clinical utility of the MVD gene signature.

In conclusion, we developed an MVD gene score using gene expression values that reflects MVD(IHC). Tumor subgroups of 12 mouse syngeneic tumor models based on MVD(IHC) and Tcell_inf_GEP correlated with the sensitivity to LEN, anti–PD-1, and their combination. Tumor subgroups of TCGA samples correlate with indications for antiangiogenic inhibitors and ICIs, highlighting the potential clinical utility of the MVD gene score together with Tcell_inf_GEP to characterize tumors of patients for precision medicine.

## Supplementary Material

Supplementary Figure S1 Antitumor activity of lenvatinib in 12 mouse syngeneic tumor models and MVD(IHC) of treatment-naïve tumor samples.

Supplementary Figure S2 Single-cell RNA-seq analysis of Hepa 1-6 mouse hepatocellular carcinoma model.

Supplementary Figure S3 Comparison of MVD gene score and other angiogenesis signatures.

Supplementary Figure S4 Distributions of TcellinfGEP in the 12 syngeneic mouse tumor models and MVD gene score and TcellinfGEP across pan-cancer dataset.

Supplementary Figure S5 Relative body weight measurements during treatment.

Supplementary Table S1 Mouse cancer cell lines.

Supplementary Table S2 Tumor types and number of human FFPE samples.

Supplementary Table S3 List of endothelial cell related genes
