# Supplementary material for: Development of a Microvessel Density Gene Signature and Its Application in Precision Medicine
Source: Cancer Res Commun. 2025 Mar 5;5(3):398–408. doi: 10.1158/2767-9764.CRC-24-0403 (PMC11880750; doi:10.1158/2767-9764.CRC-24-0403)
Supplement: Supplementary Figure S1 — Antitumor activity of lenvatinib in 12 mouse syngeneic tumor models and MVD(IHC) of treatment-naïve tumor samples. [file crc-24-0403_supplementary_figure_s1_suppsf1.docx]

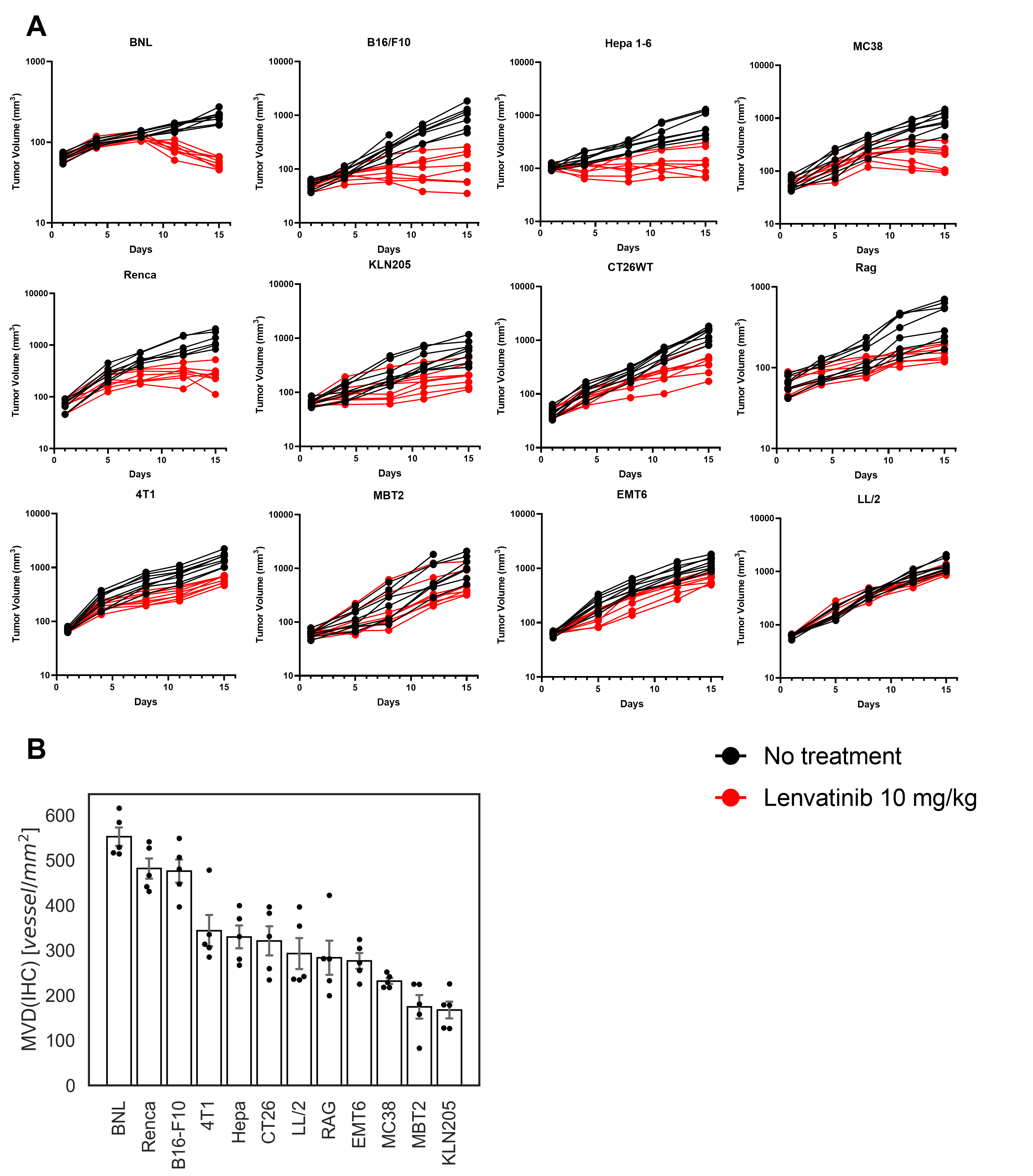


**Supplementary Figure S1. Antitumor activity of lenvatinib in 12 mouse syngeneic tumor models and MVD(IHC) of treatment-naïve tumor samples**. A, Tumor volumes of individual mice of the No treatment (black) and Lenvatinib 10 mg/kg treated groups (red). B, MVD(IHC) of treatment-naïve tumors of 12 syngeneic tumor models. Data are shown as mean ± SEM.
