# Supplementary material for: Development of a Microvessel Density Gene Signature and Its Application in Precision Medicine
Source: Cancer Res Commun. 2025 Mar 5;5(3):398–408. doi: 10.1158/2767-9764.CRC-24-0403 (PMC11880750; doi:10.1158/2767-9764.CRC-24-0403)
Supplement: Supplementary Figure S2 — Single-cell RNA-seq analysis of Hepa 1-6 mouse hepatocellular carcinoma model. [file crc-24-0403_supplementary_figure_s2_suppsf2.docx]

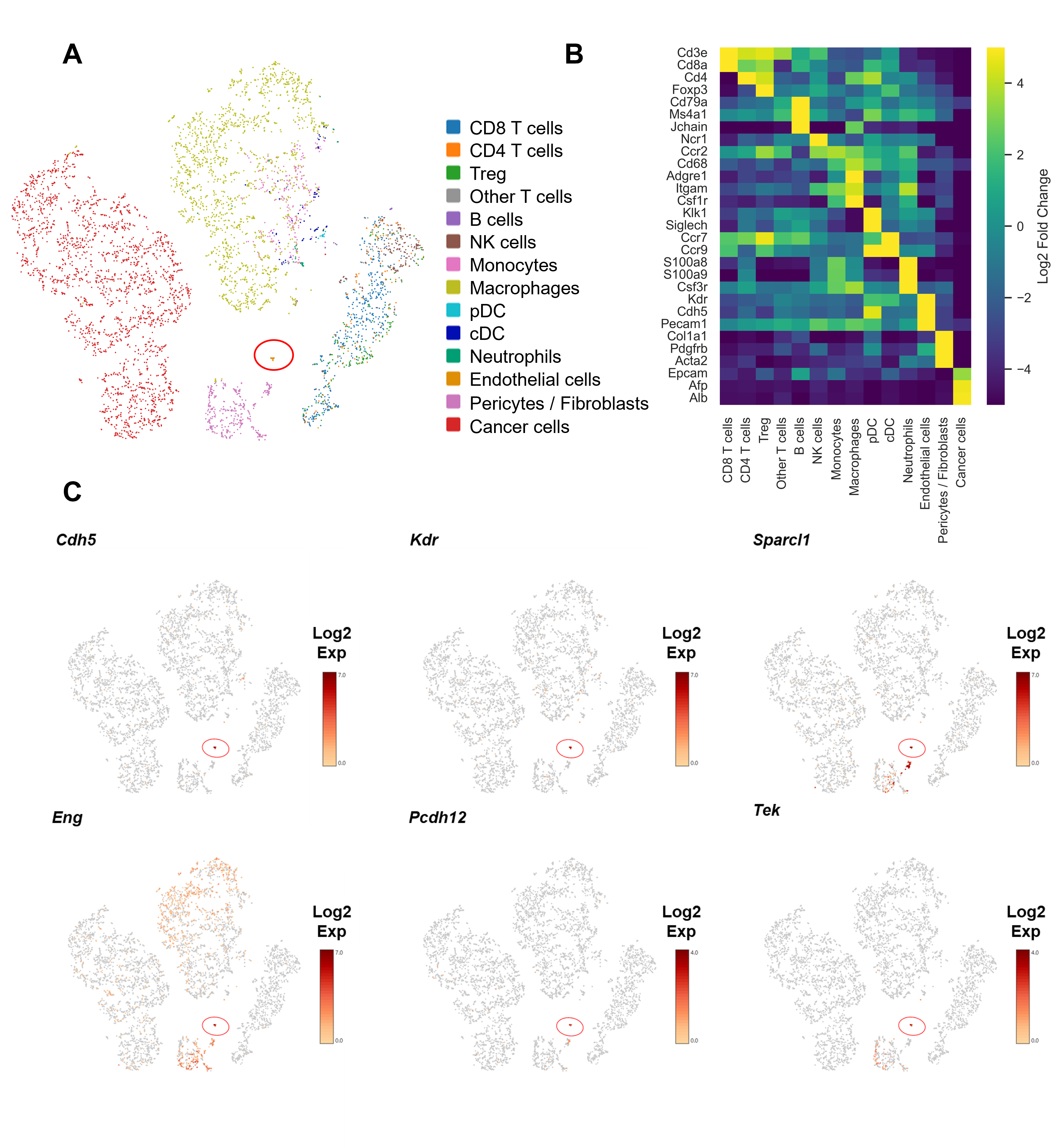


**Supplementary Figure S2. Single-cell RNA-seq analysis of Hepa 1-6 mouse hepatocellular carcinoma model.** A, Cell type annotation of 14 clusters. The cell cluster annotated as endothelial cells based on the expression levels of the marker gene *Kdr* is indicated by a red circle. B, Gene expression levels of cell type markers of the 14 clusters. C, Gene expression levels of the six MVD-related genes in scRNA-seq data of Hepa 1-6 tumors. The cell cluster annotated as endothelial cells based on the expression levels of the marker gene *Kdr* is indicated by a red circle.
