# Supplementary material for: Development of a Microvessel Density Gene Signature and Its Application in Precision Medicine
Source: Cancer Res Commun. 2025 Mar 5;5(3):398–408. doi: 10.1158/2767-9764.CRC-24-0403 (PMC11880750; doi:10.1158/2767-9764.CRC-24-0403)
Supplement: Supplementary Figure S3 — Comparison of MVD gene score and other angiogenesis signatures. [file crc-24-0403_supplementary_figure_s3_suppsf3.docx]

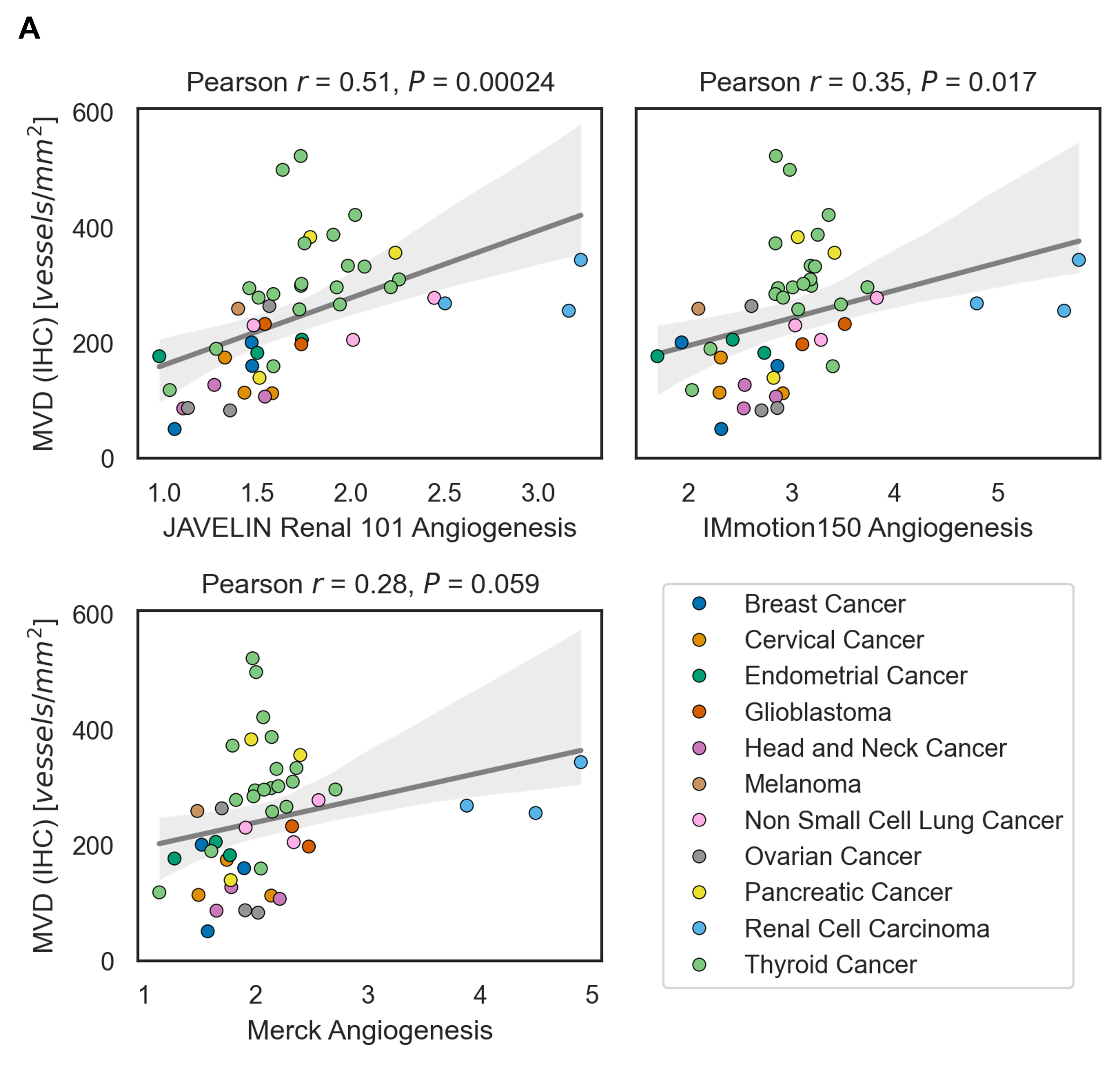

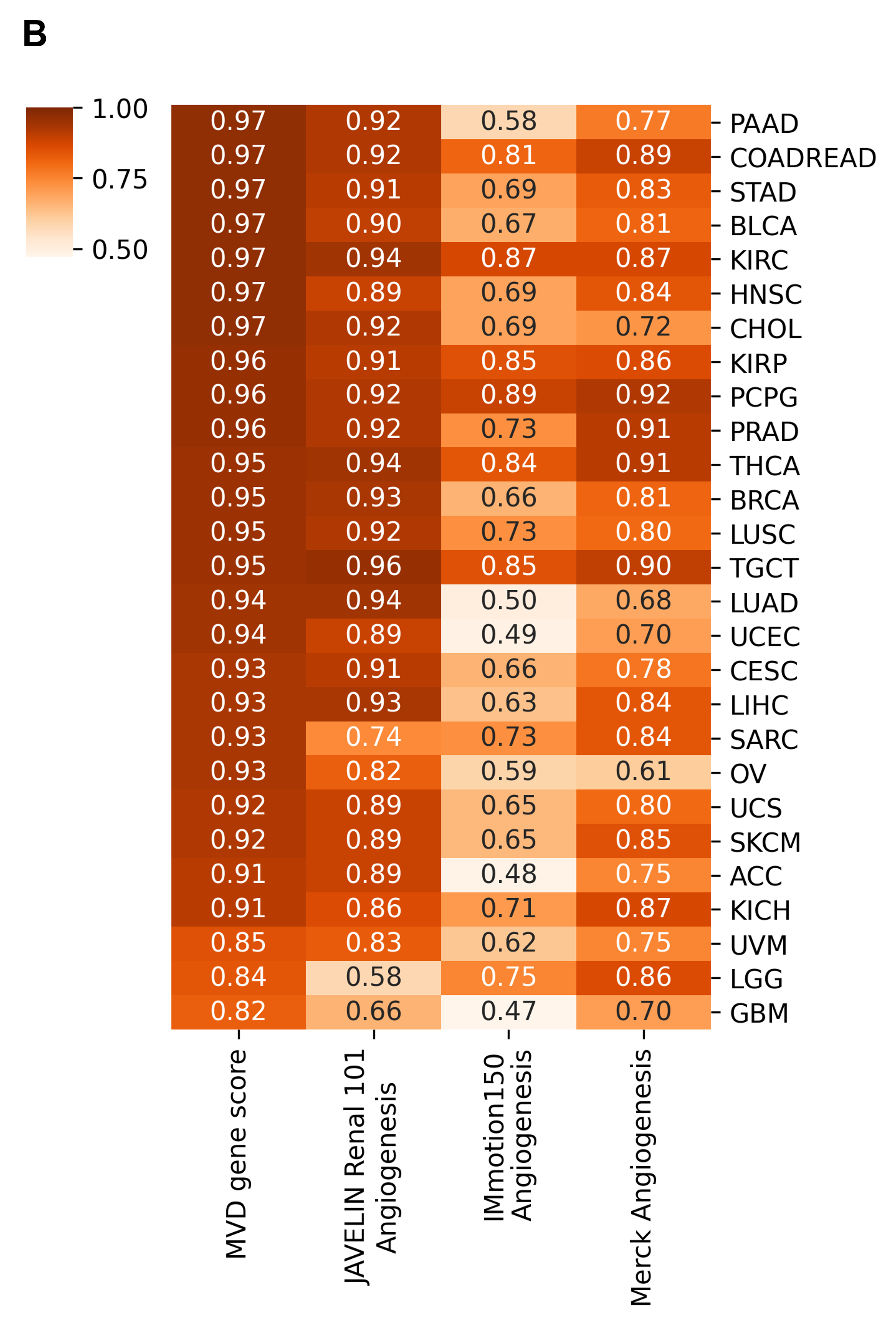


**Supplementary Figure S3. Comparison of MVD gene score and other angiogenesis signatures.** A, Correlation of MVD(IHC) and angiogenesis gene signatures from previous studies. Angiogenesis gene signatures were computed from gene expression data of commercially available FFPE samples of human tumor tissues. B, Correlation of the EC score in cell deconvolution data and angiogenesis-related gene signatures. Four angiogenesis-related gene signatures, including the MVD gene score, were computed from gene expression data of solid tumor types in the TCGA pan-cancer dataset. The correlation coefficient of each angiogenesis signature and EC score was computed for each tumor type for which cell deconvolution data were available and is shown as a heatmap. LAML: Acute Myeloid Leukemia; ACC: Adrenocortical carcinoma; BLCA: Bladder urothelial carcinoma; LGG: Brain lower grade glioma; BRCA: Breast invasive carcinoma; CESC: Cervical squamous cell carcinoma and endocervical adenocarcinoma; CHOL: Cholangiocarcinoma; COADREAD: Colon adenocarcinoma and rectum adenocarcinoma; ESCA: Esophageal carcinoma; GBM: Glioblastoma multiforme; HNSC: Head and neck squamous cell carcinoma; KICH: Kidney chromophobe; KIRC: Kidney renal clear cell carcinoma; KIRP: Kidney renal papillary cell carcinoma; LIHC: Liver hepatocellular carcinoma; LUAD: Lung adenocarcinoma; LUSC: Lung squamous cell carcinoma; DLBC: Lymphoid Neoplasm Diffuse Large B-cell Lymphoma; MESO: Mesothelioma; OV: Ovarian serous cystadenocarcinoma; PAAD: Pancreatic adenocarcinoma; PCPG: Pheochromocytoma and paraganglioma; PRAD: Prostate adenocarcinoma; SARC: Sarcoma; SKCM: Skin cutaneous melanoma; STAD: Stomach adenocarcinoma; TGCT: Testicular germ cell tumors; THYM: Thymoma; THCA: Thyroid carcinoma; UCS: Uterine carcinosarcoma; UCEC: Uterine corpus endometrial carcinoma; UVM: Uveal melanoma.
