# Supplementary material for: Development of a Microvessel Density Gene Signature and Its Application in Precision Medicine
Source: Cancer Res Commun. 2025 Mar 5;5(3):398–408. doi: 10.1158/2767-9764.CRC-24-0403 (PMC11880750; doi:10.1158/2767-9764.CRC-24-0403)
Supplement: Supplementary Figure S4 — Distributions of TcellinfGEP in the 12 syngeneic mouse tumor models and MVD gene score and TcellinfGEP across pan-cancer dataset. [file crc-24-0403_supplementary_figure_s4_suppsf4.docx]

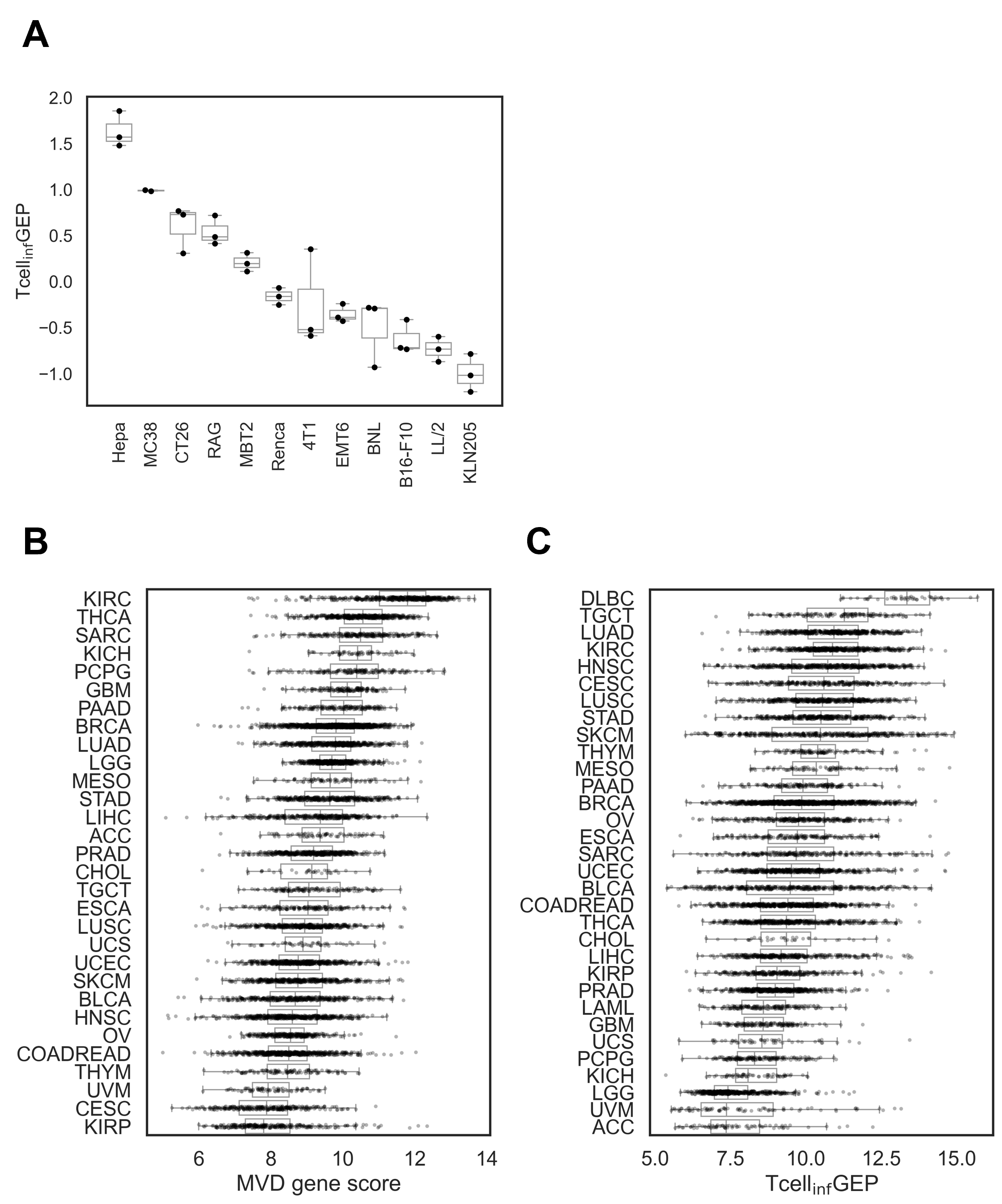


**Supplementary Figure S4. Distributions of Tcell_inf_GEP in the 12 syngeneic mouse tumor models and MVD gene score and Tcell_inf_GEP across pan-cancer dataset.** A, Tcell_inf_GEP in the 12 mouse syngeneic tumor models. Distribution of B, MVD gene score; C, Tcell_inf_GEP across the pan-cancer dataset. Gene signatures were computed using the gene expression data from TCGA pan-cancer dataset. LAML: Acute Myeloid Leukemia; ACC: Adrenocortical carcinoma; BLCA: Bladder urothelial carcinoma; LGG: Brain lower grade glioma; BRCA: Breast invasive carcinoma; CESC: Cervical squamous cell carcinoma and endocervical adenocarcinoma; CHOL: Cholangiocarcinoma; COADREAD: Colon adenocarcinoma and rectum adenocarcinoma; ESCA: Esophageal carcinoma; GBM: Glioblastoma multiforme; HNSC: Head and neck squamous cell carcinoma; KICH: Kidney chromophobe; KIRC: Kidney renal clear cell carcinoma; KIRP: Kidney renal papillary cell carcinoma; LIHC: Liver hepatocellular carcinoma; LUAD: Lung adenocarcinoma; LUSC: Lung squamous cell carcinoma; DLBC: Lymphoid Neoplasm Diffuse Large B-cell Lymphoma; MESO: Mesothelioma; OV: Ovarian serous cystadenocarcinoma; PAAD: Pancreatic adenocarcinoma; PCPG: Pheochromocytoma and paraganglioma; PRAD: Prostate adenocarcinoma; SARC: Sarcoma; SKCM: Skin cutaneous melanoma; STAD: Stomach adenocarcinoma; TGCT: Testicular germ cell tumors; THYM: Thymoma; THCA: Thyroid carcinoma; UCS: Uterine carcinosarcoma; UCEC: Uterine corpus endometrial carcinoma; UVM: Uveal melanoma.
