# Supplementary material for: Development of a Microvessel Density Gene Signature and Its Application in Precision Medicine
Source: Cancer Res Commun. 2025 Mar 5;5(3):398–408. doi: 10.1158/2767-9764.CRC-24-0403 (PMC11880750; doi:10.1158/2767-9764.CRC-24-0403)
Supplement: Supplementary Figure S5 — Relative body weight measurements during treatment. [file crc-24-0403_supplementary_figure_s5_suppsf5.docx]

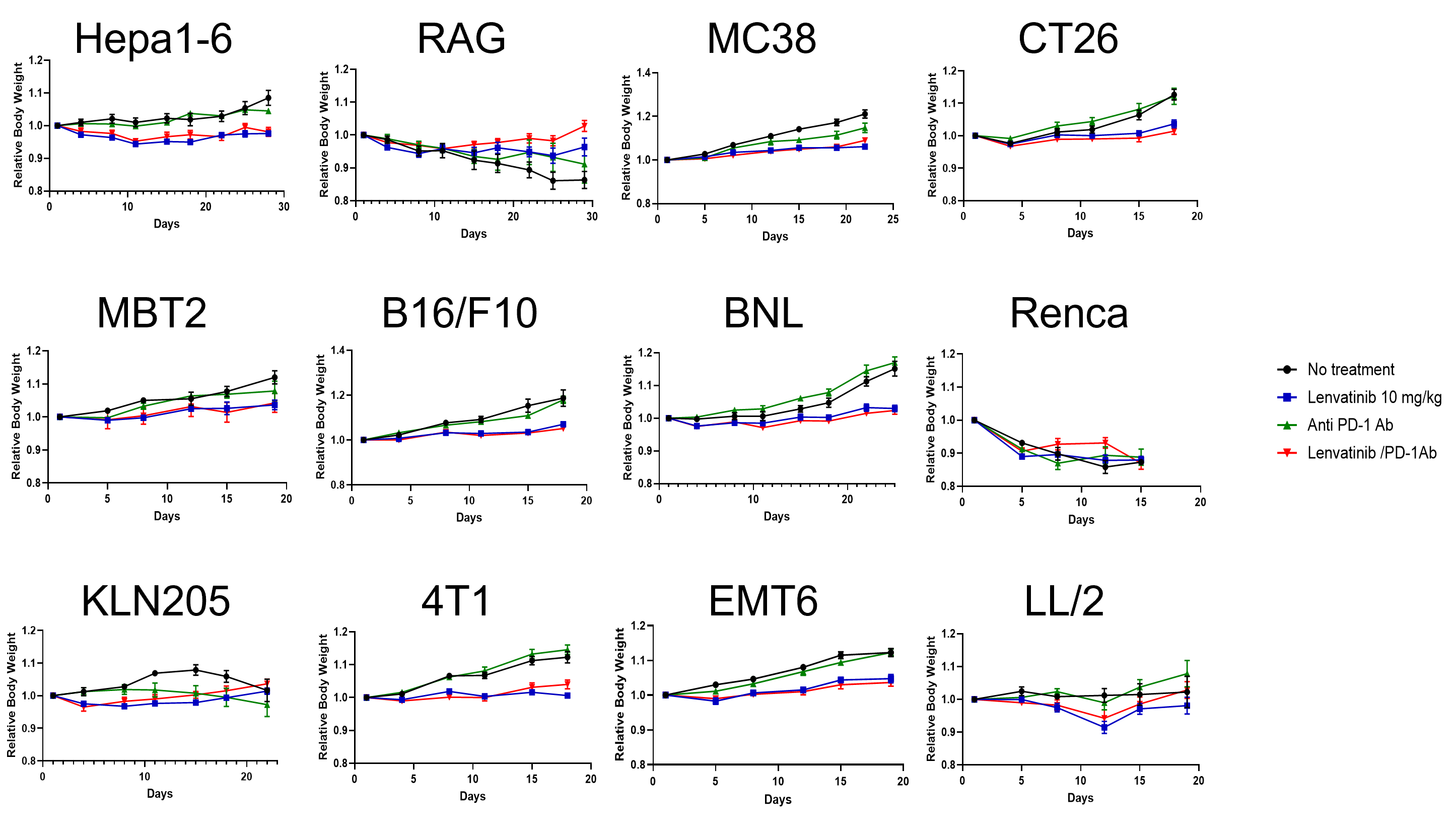


**Supplementary Figure S5.** **Relative body weight measurements during treatment.** Relative body weight of No treatment, Lenvatinib 10 mg/kg, anti-PD-1 Ab 200 μg/head, and their combination groups. Data are shown as mean ± SEM (n = 8).
