# Supplementary material for: Development of a Microvessel Density Gene Signature and Its Application in Precision Medicine
Source: Cancer Res Commun. 2025 Mar 5;5(3):398–408. doi: 10.1158/2767-9764.CRC-24-0403 (PMC11880750; doi:10.1158/2767-9764.CRC-24-0403)
Supplement: Supplementary Table S1 — Mouse cancer cell lines. [file crc-24-0403_supplementary_table_s1_suppst1.docx]

| **Mouse cancer cell name** | | **Purchased from** | **Subcutaneous transplantation to** |
| --- | --- | --- | --- |
| **Hepa 1-6** | HCC | ATCC | C57L/J, male |
| **BNL 1ME A.7R.1** | HCC | ATCC | BALB/cAnNCrlCrlj, male |
| **RAG** | RCC | ATCC | BALB/cAnNCrlCrlj, female |
| **Renca** | RCC | ATCC | BALB/cAnNCrlCrlj, female |
| **KLN205** | Lung cancer | ATCC | DBA/2NCrl, female |
| **LL/2** | Lung cancer | ATCC | C57BL/6JCrl, female |
| **CT26.WT** | CRC | ATCC | BALB/cAnNCrlCrlj, female |
| **MC38** | CRC | Karefast | C57BL/6NCrl, female |
| **EMT6** | Breast cancer | ATCC | BALB/cAnNCrlCrlj, female |
| **4T1** | Breast cancer | ATCC | BALB/cAnNCrlCrlj, female |
| **MBT2** | Bladder cancer | JCRB | C3H/HeNCrl, female |
| **B16-F10** | Melanoma | ATCC | C57BL/6NCrl, female |

**Supplementary Table S1. Mouse cancer cell lines**
