# Supplementary material for: Development of a Microvessel Density Gene Signature and Its Application in Precision Medicine
Source: Cancer Res Commun. 2025 Mar 5;5(3):398–408. doi: 10.1158/2767-9764.CRC-24-0403 (PMC11880750; doi:10.1158/2767-9764.CRC-24-0403)
Supplement: Supplementary Table S2 — Tumor types and number of human FFPE samples. [file crc-24-0403_supplementary_table_s2_suppst2.docx]

| **Clinical diagnosis of human tumor samples** | **#Samples** |
| --- | --- |
| Thyroid Cancer | 20 |
| Breast Cancer | 3 |
| Cervical Cancer | 3 |
| Endometrial Cancer | 3 |
| Glioblastoma | 2 |
| Head and Neck Cancer | 3 |
| Melanoma | 1 |
| Non-small Cell Lung Cancer | 3 |
| Ovarian Cancer | 3 |
| Pancreatic Cancer | 3 |
| Renal Cell Carcinoma | 3 |

**Supplementary Table S2. Tumor types and number of human FFPE samples**
